# Clinical outcomes and prognostic factors of bronchiectasis rheumatoid overlap syndrome: A multi-institution cohort study

**DOI:** 10.3389/fmed.2022.1004550

**Published:** 2022-10-13

**Authors:** Horng-Chyuan Lin, Hung-Yu Huang, Chun-Yu Lin, Yueh-Fu Fang, Chiung-Hung Lin, Yu-Tung Huang, Chiung-Hsin Chang, Chun-Hua Wang, Jhen-Ling Huang, Ting-Wei Liao, Meng-Heng Hsieh

**Affiliations:** ^1^Department of Thoracic Medicine, Chang Gung Memorial Hospital, Taipei, Taiwan; ^2^College of Medicine, Chang Gung University, Taoyuan, Taiwan; ^3^Department of Thoracic Medicine, New Taipei City Municipal Tucheng Hospital, Chang Gung Medical Foundation, New Taipei City, Taiwan; ^4^Center for Big Data Analytics and Statistics, Chang Gung Memorial Hospital, Taoyuan, Taiwan

**Keywords:** bronchiectasis rheumatoid overlap syndrome, DMARD, infection, biological agents, mortality

## Abstract

The information regarding bronchiectasis with RA (BROS) is limited in Asia. The objective of this study was to investigate the clinical characteristics and outcomes of BROS in Taiwan. This multi-institute cohort study included patients with BROS from January 2006 to December 2017. The clinical, functional and microbiological data of these patients were retrieved from the Chang Gung Research Database. Respiratory failure and mortality were the primary outcomes. Severe exacerbation was defined as bronchiectasis- related hospitalizations or emergency department visits. A total of 343 patients with BROS were identified. One hundred and eight patients had severe exacerbation and exhibited significantly more previous exacerbations, a lower FEV1 and higher BACI score (11.1 vs. 7.5) than patients without severe exacerbation. The most prevalent species in sputum were Non-tuberculous mycobacteria (NTM) (14.8 %), *Pseudomonas aeruginosa* (14.2 %), and *fungus* (5.9%). 68.8% of BROS patients used disease modifying antirheumatic drugs (DMARD), 7.9% used biological DMARD. NTM and tuberculosis infection rates were higher in bDMARD group compared with nbDMARD group and others. Overall, the 3-year respiratory failure rate and mortality rate were 14.6 and 25.7% respectively. Patients with RA diagnosed before bronchiectasis had a significantly higher cumulative incidence of mortality in a 3-year follow-up than those with RA diagnosed after bronchiectasis. In Cox regression, age, higher RF value and systemic steroid use were independent risk factors for mortality in BROS. BROS patients with severe exacerbation had a high mortality rate in Taiwan. bDMARD is associated with a trend of increased risk of NTM and TB infections.

## Introduction

Bronchiectasis is characterized by permanent dilatation of airways, recurrent pulmonary infection and systemic inflammation (1). Comorbidities are prevalent in patients with bronchiectasis and contribute to increased mortality (2). Bronchiectasis with comorbidities can be evaluated using bronchiectasis etiology comorbidity index (BACI) to predict clinical outcomes (2, 3).

Connective tissue disease is a common comorbidity of bronchiectasis, accounting for 12% of patients with bronchiectasis in a European cohort and 6% of those in a cohort from Korea (2, 4). Rheumatoid arthritis (RA) is one of the most common autoimmune diseases associated with bronchiectasis (5). Studies have reported that 3–12% of RA patients had symptomatic bronchiectasis and the prevalence of RA in bronchiectasis is variable in previous studies (6, 7). RA can be considered an etiology or comorbidity of bronchiectasis because the symptoms of RA may precede or succeed the diagnosis of bronchiectasis (7).

Bronchiectasis rheumatoid overlap syndrome (BROS) is associated with worse survival outcome than idiopathic bronchiectasis (6). The 4-year mortality rate of BROS was reported to be 18% in the United Kingdom (6). There are several clinical features in BROS reported in previous studies and may be associated with excess mortality. One study observed higher bronchiectasis severity index (BSI) scores in BROS than in idiopathic bronchiectasis (6). Because BSI score is a predictor of mortality (8), the higher BSI score for BROS indicates more severe clinical status. Another study found patients with BROS to have higher levels of RA autoantibodies and more severe RA compared with those with RA alone (9). Patients with RA have a relatively high risk of infections owing to autoantibodies and immune modulation therapy (10–13). The infection risk of RA may in turn aggravate the vicious cycle of bronchiectasis in patients with BROS. Bronchiectasis can be diagnosed before or after RA diagnosis, and whether the sequence of diagnosis can affect clinical outcomes is not known.

Few studies have reported the epidemiology or clinical outcomes of BROS in Asia. Furthermore, no study has investigated the prognostic factors for BROS. Accordingly, the present study surveyed the clinical manifestations and prognosis of BROS and investigated risk factors associated with respiratory failure and mortality in an Asian cohort.

## Methods

### Study population

This multi-institutional cohort study used the Chang Gung Research Database (CGRD). The CGRD contains electronic medical records collected from the Chang Gung Memorial Hospital system, which comprises three medical centers and four regional hospitals. Detailed information regarding the CGRD has been provided in previous studies (14, 15). The bronchiectasis cohort included patients with at least two bronchiectasis diagnoses (*International Classification of Diseases, Ninth Revision, Clinical Modification* [*ICD-9-CM*] code 494.0 or 494.1, or *International Classification of Diseases, Tenth Revision, Clinical Modification* [*ICD-10-CM*] code J47) during outpatient visits or one diagnosis during hospitalization between January 1, 2006, and December 31, 2017 (16). These patients have received high resolution computed tomography, which was independently reviewed by a radiologist. BROS was defined as a diagnosis of RA—made either before or after the diagnosis of bronchiectasis—in the bronchiectasis cohort (7). The diagnosis of RA was identified using *ICD-9-CM* code 714.0 or *ICD-10-CM* code M05.70–M06.09, M06.20–M06.39, M06.80–M06.89, or M06.9 (17, 18). The Institutional Review Board of Chang Gung Memorial Hospital approved this study (IRB number: 201800712B0C502).

The index date was defined as the date when the patients were diagnosed to have both bronchiectasis and RA. Bronchiectasis or RA could both be the first diagnosis and the gap year between the first diagnosis and confirmed BROS was recorded. Because RA is a recognized etiology of bronchiectasis, those patients with concurrent diagnosis of both bronchiectasis and RA within 1 month were included in the RA first group. The BROS patients were followed up for 3 years since the index data and the study outcomes were recorded during the 3-year period.

### Main outcomes

The primary outcomes were 3-year overall mortality and respiratory failure rates after diagnosis of BROS. Acute respiratory failure was defined according to the ICD-9-CM code 518.81 or 518.82 or ICD-10-CM code J96.0 with mechanical ventilator use (19). The causes of death were retrieved from health insurance database of Taiwan. Severe exacerbation was defined as bronchiectasis- related hospitalizations or emergency department visits, which included hemoptysis and pneumonia (ICD-9-CM codes (481, 482, 483, 485, and 486) with antibiotic use more than 1 week (20, 21). Clinic exacerbation was defined as the need for antibiotics in a clinic for the deterioration in respiratory symptoms (20).

### Clinical parameters

This study retrieved demographic data; laboratory, microbiology, and pulmonary function reports; and medical records from the CGRD. The BACI score was calculated on the basis of comorbidities observed from diagnoses in the CGRD (2, 3). This study also collected sputum microbiology reports. The sputum could be taken either in stable state or during exacerbation. Some patients may not have sputum when the diagnosis of BROS was made and sputum cultures for both bacteria and mycobacteria were performed for productive cough or deterioration of respiratory symptoms during follow-up. Chronic infection was defined as isolation of the same pathogen in two or more cultures, at least 3 months apart within 12 months (22). Previous exacerbation (1-year) was defined as the number of exacerbations recorded during the 1-year period before diagnosis of BROS (the index date). Autoimmune markers of RA included rheumatoid factor (RF) and anticyclic citrullinated peptide antibody (anti-CCP). A pulmonary function test was performed using a spirometer in accordance with the criteria of the American Thoracic Society and the European Respiratory Society (23). Medications for RA treatment included systemic corticosteroids, non-biological disease-modifying antirheumatic drugs (nbDMARDs), and biological disease-modifying antirheumatic drugs (bDMARDs). In Taiwan, patients with RA can be reimbursed for the use of bDMARDs if they fulfill the criteria of continuously active RA with a disease activity score (DAS)-28 value of >5.1 and no significant efficacy after at least two kinds of nbDMARD administration (24). This study recorded medications used more than 1 month during out-patient department follow-up.

### Statistical analysis

The chi-square (χ2) and Fisher exact tests were conducted for dichotomous variables, the independent t-test was performed for normally distributed continuous variables, and the Mann–Whitney U test was used for non-normally distributed continuous data. A two-sided *p*-value of <0.05 was considered statistically significant. In addition, a univariate descriptive analysis was conducted to identify risk factors for respiratory failure and mortality in patients with BROS. Variables with a significance level of p < 0.05 were selected. Subsequently, a multivariate Cox proportional hazards regression with stepwise method (α_in_ = 0.25 α_out_ = 0.15) was used to identify independent risk factors. All statistical analyses were performed using SAS software (version 9.4; SAS Institute, Cary, NC, USA).

## Results

There were 10,213 patients with bronchiectasis during the study period and overall, 343 patients with BROS were identified in the study cohort. Their mean age was 65 years, and 24% of them had a history of emergency room visits or hospitalization. On average, 13% of the patients had chronic *Pseudomonas aeruginosa* infection. The mean BACI score was 8.6 ± 5.4.

Table 1 presents a comparison of patients with BROS with or without severe exacerbations. Generally, the patients with BROS with severe exacerbations were considerably older, were more often men, had higher BACI scores, had smaller forced expiratory volumes in 1 s, and had more previous exacerbations. Furthermore, the patients with BROS with severe exacerbations had higher CRP and ESR levels. The RF and anti-CCP levels were similar in both groups, and 62.9% of the patients with BROS with severe exacerbations had respiratory failure. The 3-year mortality rates were 25.6% for the patients with BROS and 81.5% for those with severe exacerbations. The most common pathogens were *P. aeruginosa*, non-tuberculosis mycobacterium (NTM), *Stenotrophomonas maltophilia, Haemophilus influenzae*, and fungi (Table 2). Positive rates of sputum microbiology of BROS with or without severe exacerbation were in Supplementary Table S2. Pulmonary infection (31.8%), cardiovascular complications (15.9%), RA-associated complications (10.2%), and malignancy (7.9%) were the primary causes of mortality (Table 3). In this study, 90% of RA markers and pulmonary function tests were measured within 3 months of the index date. The timing of the sputum collection was 30% within 3 months of the index date, 60% within 1 year after index date and other sputum tests were during 3 years follow-up after diagnosis of BROS. As for systemic steroid usage, the mean daily dosage was 86.7 mg cortisone for general group and 112 mg cortisone for severe group. The duration of systemic steroid use was 18% within 3 months, 22% between 3 and 12 months, and 60% more than 1 year.

**Table 1:** Characteristics and outcomes of BROS with or without severe exacerbation.

	**Total**	**General^#^**	**Severe**	***p*-value**
	**(*n* = 343)**	**(*n* = 235)**	**(*n* = 108)**	
Age	65.4 ± 11.5	63.1 ± 11.2	70.6 ± 10.6	< .0001
Gender (female)	264 (76.9)	188 (80.0)	76 (70.4)	0.049
**Pulmonary function**				0.007
FEV1 > 80%	82 (35.0)	68 (40.0)	14 (21.8)	
FEV1 50–80%	40 (17.1)	26 (15.3)	14 (21.8)	
FEV1 < 50%	21 (8.9)	10 (5.9)	11 (17.2)	
FVC < 80%	91 (38.9)	66 (38.8)	25 (39.1)	
BACI index	8.6 ± 5.4	7.5 ± 4.7	11.1 ± 6.2	< 0.0001
**Comorbidity**				
Solid malignancy	12 (3.5)	3 (1.3)	9 (8.3)	0.002
Liver disease	60 (17.5)	43 (18.3)	17 (15.7)	0.563
Diabetes	61 (17.8)	27 (11.5)	34 (31.5)	< 0.0001
Chronic renal disease	49 (14.3)	28 (11.9)	21 (19.4)	0.064
Hematological malignancy	11 (3.2)	4 (1.7)	7 (6.5)	0.041
Stroke	46 (13.4)	19 (8.1)	27 (25.0)	< 0.0001
**RA markers**				
Anti-CCP	78.8 ± 129.6	77.2 ± 134.3	82.9 ± 124.4	0.321
RF value	199.3 ± 637.6	154.1 ± 259.8	307.0 ± 1100.3	0.145
C-reactive protein	37.5 ± 57.5	26.5 ± 47.1	58.9 ± 69.1	< 0.0001
ESR	37.9 ± 29.8	35.0 ± 28.6	44.8 ± 31.6	0.009
Gap years*	3.4 ± 3.1	3.3 ± 2.9	3.7 ± 3.6	0.882
**Medication**				
Systemic steroid	271 (79.0)	169 (71.9)	102 (94.4)	< 0.0001
Steroid dosage^&^	102.8 (97.1)	86.8 (87.9)	112.0 (101.5)	0.292
DMARD	237 (69.0)	164 (69.8)	73 (67.5)	0.706
Biologicals	27 (7.8)	20 (8.5)	7 (6.5)	0.517
Previous AE (1-year)	1.0 ± 2.0	0.7 ± 1.7	1.7 ± 2.5	< 0.0001
Hospitalization	0.3 ± 0.7	0.2 ± 0.5	0.5 ± 1.1	< 0.0001
Emergency room visit	0.5 ± 1.2	0.3 ± 1.0	0.9 ± 1.5	< 0.0001
Clinic	0.3 ± 1.3	0.3 ± 1.2	0.4 ± 1.5	0.171
Respiratory failure (1-year)	41 (12.0)	0 (0)	41 (37.9)	< 0.0001
Respiratory failure (3-year)	68 (19.8)	0 (0)	68 (62.9)	< 0.0001
Mortality (1-year)	41 (12.0)	0 (0)	41 (37.9)	< 0.0001
Mortality (3-year)	88 (25.7)	0 (0)	88 (81.5)	< 0.0001

AE, acute exacerbation; Anti-CCP, anti-cyclic citrullinated peptide antibody; BROS, Bronchiectasis Rheumatoid Overlap Syndrome; ESR, erythrocyte sedimentation rate; FEV1, forced expiratory volume in 1 s; FVC, forced vital capacity, RA, rheumatoid arthritis.

^*^Gap between RA & bronchiectasis, years.

^#^ General group: BROS patients without severe exacerbation.

^&^ Equivalent to daily cortisone doses (mg).

**Table 2:** Sputum microbiology of BROS with or without severe exacerbation during 3 years follow-up.

**General**		**Severe**	
Microbiology	*N* (%)	Microbiology	*N* (%)
NTM	40 (22.1)	*Pseudomonas aeruginosa*	37 (17.5)
Negative	26 (14.4)	NTM	18 (8.5)
*Pseudomonas aeruginosa*	19 (10.5)	Negative	17 (8.1)
*Haemophillus influenzae*	14 (7.7)	*Stenotrophomonas*	15 (7.1)
Fungus	12 (6.6)	*Fungus*	11 (5.2)
*Staphylococcus aureus*	9 (4.9)	*Acinetobacter baumannii*	10 (4.7)
*Streptococcus pneumoniae*	8 (4.4)	*Staphylococcus aureus*	10 (4.7)
*Klebsiella pneumoniae*	6 (3.3)	*E. coli*	9 (4.3)
Tuberculosis	4 (2.1)	*Klebsiella pneumoniae*	9 (4.3)

BROS, bronchiectasis rheumatoid overlap syndrome; NTM, non-tuberculosis mycobacteria.

**Table 3:** Characteristics and outcomes of BROS grouped with diagnosis chronology.

	**Total**	**RA first**	**BR first**	***p*-value**
	**(*n* = 343)**	**(*n* = 230)**	**(*n* = 113)**	
Age	65.4 ± 11.5	65.7 ± 11.6	64.8 ± 11.2	0.471
**Gender**				0.780
Female	264 (76.9)	176 (76.5)	88 (77.9)	
Male	79 (23.0)	54 (23.5)	25 (22.1)	
**Pulmonary function**				0.690
FEV1 > 80%	82 (35.0)	52 (34.9)	30 (35.3)	
FEV1 50–80%	40 (17.1)	27 (18.1)	13 (15.3)	
FEV1 < 50%	21 (8.9)	11 (7.3)	10 (11.7)	
FVC < 80%	91 (38.9)	59 (39.6)	32 (37.6)	
BACI index	8.6 ± 5.4	8.9 ± 5.2	8.2 ± 5.9	0.077
**Comorbidity**				
Solid malignancy	12 (3.5)	9 (3.9)	3 (2.6)	0.757
Liver disease	60 (17.5)	37 (16.1)	23 (20.4)	0.328
Diabetes	61 (17.8)	41 (17.8)	20 (17.7)	0.977
Chronic renal disease	49 (14.3)	35 (15.2)	14 (12.4)	0.482
Hematological malignancy	11 (3.2)	10 (4.4)	1 (0.8)	0.110
Stroke	46 (13.4)	32 (13.9)	14 (12.4)	0.697
**RA markers**				
Anti-CCP	78.9 ± 129.6	84.4 ± 129.9	68.3 ± 134.7	0.637
RF value	199.3 ± 637.6	239.0 ± 754.0	111.4 ± 197.1	0.043
C-reactive protein	37.5 ± 57.5	40.9 ± 62.7	30.2 ± 43.7	0.128
ESR	37.9 ± 29.8	38.4 ± 31.3	36.8 ± 26.5	0.835
Gap years	3.4 ± 3.1	3.5 ± 3.2	3.4 ± 2.9	0.728
**Medication**				
Systemic steroid	271 (79.0)	192 (83.5)	79 (69.9)	0.004
DMARD	237 (69.0)	172 (74.7)	65 (57.5)	0.001
Biologicals	27 (7.8)	22 (9.6)	5 (4.4)	0.097
Previous AE (1-year)	1.0 ± 2.0	0.9 ± 1.8	1.2 ± 2.4	0.921
Hospitalization	0.3 ± 0.8	0.2 ± 0.6	0.4 ± 1.0	0.371
Emergency room visit	0.5 ± 1.2	0.5 ± 1.1	0.6 ± 1.4	0.695
Clinic	0.3 ± 1.3	0.3 ± 1.2	0.4 ± 1.5	0.990
Respiratory failure (1-year)	41 (11.9)	33 (14.4)	8 (7.1)	0.051
Respiratory failure (3-year)	68 (19.8)	52 (22.6)	16 (14.2)	0.065
Mortality (1-year)	41 (11.9)	33 (14.4)	8 (7.1)	0.051
Mortality (3-year)	88 (25.7)	70 (30.4)	18 (15.9)	0.004
**Death cause**				0.960
Cardiovascular	14 (15.9)	11 (15.7)	3 (16.7)	
Lung infection	28 (31.8)	23 (32.9)	5 (27.8)	
Malignancy	7 (7.9)	5 (7.1)	2 (11.1)	
RA associated	9 (10.2)	7 (10.0)	2 (11.1)	
Other	30 (34.1)	24 (34.3)	6 (33.3)	

AE, acute exacerbation; Anti-CCP, anti-cyclic citrullinated peptide antibody; BROS, bronchiectasis rheumatoid overlap syndrome; DMARD, disease-modifying antirheumatic drugs; ESR, erythrocyte sedimentation rate; FEV1, forced expiratory volume in 1 s; FVC, forced vital capacity; RA, rheumatoid arthritis.

*Gap between RA & bronchiectasis, years.

**Figure 1:**
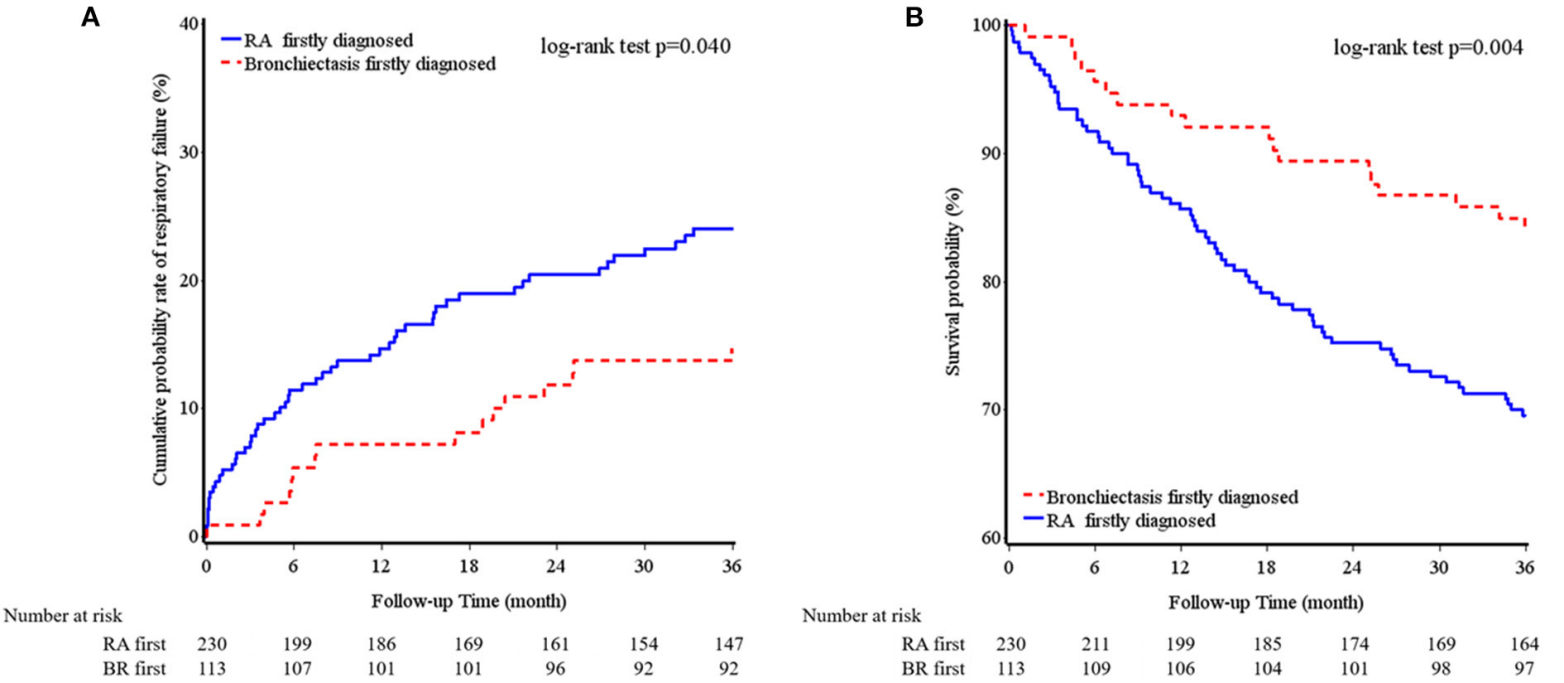
Cumulative incidence of respiratory failure **(A)** and overall survival **(B)** of bronchiectasis and rheumatoid arthritis chronology within a 3-year follow-up period.

Table 3 provides a comparison between the patients with diagnoses of bronchiectasis before and after RA. There were 24 patients with the diagnoses of RA and bronchiectasis within 1 month. Because RA is a recognized etiology of bronchiectasis, these 24 patients were included in the RA first group. Both groups had a similar average age and similar BACI scores and previous exacerbations. The patients with RA diagnosed before bronchiectasis had significantly higher RF levels and greater systemic steroid and nbDMARD use than did those with RA diagnosed after bronchiectasis. Furthermore, the patients with RA diagnosed before bronchiectasis had a considerably higher cumulative incidence of mortality within a 3-year follow-up period than did those with RA diagnosed after bronchiectasis (Figure 1). Percentage of missing data of clinical characters of BROS grouped with diagnosis chronology were in Supplementary Table S3.

Supplementary Table S1 also provides a comparison of the RA medication groups; as shown, the bDMARD and nbDMARD groups had the highest RF, CRP, and ESR levels. The 3-year respiratory failure and mortality rates were similar across all groups. The NTM and tuberculosis infection rates were higher in the bDMARD group than in the nbDMARD group and the other groups (Supplementary Table S1). All tuberculosis infection were after the use of nbDMARD and bDMARD. NTM infection were also after the use of nbDMARD and bDMARD, except those in the others group. The duration of nbDMARD use was 10% within 3 months, 15% between 3 and 12 months, and 75% more than 1 year. The duration of bDMARD use was 50% within 3 months, 25% between 3 and 12 months, and 25% more than 1 year.

The Cox regression results revealed that age, diabetes, hematological malignancy, systemic steroid use, previous exacerbations, and bDMARD use were risk factors for respiratory failure after multivariable adjustment (Table 4). In addition, old age, a high RF level, and systemic steroid use were independently associated with increased mortality (Table 4).

**Table 4:** Stepwise cox regression model of 3-year mortality and respiratory failure.

**Variable**	**Hazard ratio**	**95% CI**	***p*-value**
**Death (3-year)**				
Age, years	1.05	1.02–1.08	< 0.001
Malignancy	1.96	0.86–4.45	0.110
Diabetes mellitus	1.58	0.88–2.82	0.122
RF value	1.000232	1.000039–1.000424	0.018
Systemic steroid	4.32	1.33–14.04	0.015
Previous ER visit (1-year)	1.66	0.95–2.91	0.076
**Respiratory failure (3-year)**				
Age, years	1.02	1.00–1.05	0.033
Diabetes mellitus	1.89	1.12–3.19	0.017
Systemic steroid	12.46	1.71–90.90	0.013
Biologic agents use	3.65	1.11–11.95	0.032
Previous hospitalization (1-year)	2.09	1.24–3.53	0.006
Previous clinic AE (1-year)	1.84	0.93–3.65	0.080

AE, acute exacerbation.

## Discussion

This study found that 31% of the patients with BROS had severe exacerbations and that 20% of these patients developed respiratory failure; the overall 3-year mortality rate was 25%. The patients with RA diagnosed before bronchiectasis had a considerably higher cumulative incidence of mortality within a 3-year follow-up period than did those with RA diagnosed after bronchiectasis. In addition, bDMARD and nbDMARD use appeared to increase the risk of tuberculosis and NTM infection in the patients with BROS.

Few studies have reported longitudinal outcomes of BROS. In the CGRD cohort, 31% of the patients with BROS exhibited severe exacerbations during the 3-year follow-up period. BROS patients with severe exacerbation were older, more male and had worse lung function, higher BACI score and more previous exacerbation rates. Old age, a higher RF level, diabetes, previous exacerbations, systemic steroid use, and combined bDMARD and nbDMARD use were risk factors for respiratory failure.

BROS is associated with worse survival outcome than idiopathic bronchiectasis (6).

In the United Kingdom, the 4-year mortality rate is 9.3% in idiopathic BR, 18% for RA, and 28.5% for BCOS (6). In the present study, the overall 3-year mortality rate was 25.7%, but for the patients with severe exacerbations, the 3-year mortality rate was as high as 81.5%. BROS exhibits twice the mortality risk of idiopathic bronchiectasis and also a higher BSI score (6). However, no previous study has investigated the risk factors for poorer outcomes in BROS. The present study found that old age, a higher RF level, and systemic steroid use were associated with increased mortality in patients with BROS. In addition, RA, RF, and anti-CCP were reported to be associated with poorer bDMARD control (25). Compared with RA alone, BROS exhibits a higher prevalence of positive serum autoantibody levels, infammatory markers, and joint involvement (5). The possible factors behind the poorer outcomes in BROS than in RA alone are severe immune responses and systemic infammation (9). Furthermore, this study revealed that pulmonary infections (31.8%), cardiovascular complications (15.9%), RA-associated complications (10.2%), and malignancy (7.9%) were the primary causes of mortality. This finding is noteworthy because pulmonary infections are common in patients with bronchiectasis, and RA has been found to lead to a relatively high incidence of cardiovascular death (26–28).

The diagnosis of bronchiectasis can occur either before or after RA (7). In this study, the mean gap is 3.4 years with standard deviation of 3.1 years. The gap between RA and bronchiectasis ranged from 5 years to 11 years in several patients of this study. Previous studies reported the gaps between RA and bronchiectasis from 19 to 24.7 years (29–31). These studies were small scale and mostly reported before 2001 (29–31). CT scans may not be performed widely and lead to delayed diagnosis of bronchiectasis after RA. Besides, respiratory symptoms precede joint symptoms in a large portion of BROS patients (5). RA biomarkers (RF and anti-CCP) is suggested to be measured when respiratory symptoms are present to decrease the duration between diagnosis of bronchiectasis and RA. Bacterial antigen from recurrent lung infection may trigger autoimmunity (32). In addition, positive levels of RF and anti-CCP antibodies are associated with a higher risk of RA in patients with bronchiectasis (5). A previous study reported that 50% of a group of patients with bronchiectasis with a positive RF or anti-CCP level developed RA within 1 year (5). More studies will be needed to investigate the gap between RA and bronchiectasis. Respiratory symptoms such as dyspnea, productive cough and hemoptysis seemed unaffected by the timing of RA diagnosis (7). The present study indicated that the patients with RA diagnosed before bronchiectasis had higher RF levels and greater systemic steroid and nbDMARD use; however, their BACI scores and previous exacerbation rates were similar to those of the patients with RA diagnosed after bronchiectasis. This study also revealed that the timing of RA diagnosis influenced clinical outcomes. The patients with RA diagnosed before bronchiectasis had a considerably higher cumulative incidence of mortality within a 3-year follow-up period than did those with RA diagnosed after bronchiectasis. We also observed that RA severity seemed to be higher in the patients with RA diagnosed before bronchiectasis because of the higher rates of immunosuppressive therapy use. Nevertheless, the influence of the diagnosis sequence on the clinical outcomes requires further investigation.

Biologic immune modulation treatment is used to treat patients with RA who inadequately respond to nb-DMARDs and bDMARDs is associated with an increased risk of infections in RA, including general infection, tuberculosis and serious infection (12, 13). A previous study including 47 patients with BROS observed frequent pulmonary infections after the administration of bDMARDs. Although the clinical impact of bDMARD treatment on outcomes of BROS remain largely unknown, the present study demonstrated that the patients with BROS and bDMARD use had higher previous exacerbation rates and a longer duration between the diagnoses of RA and bronchiectasis. The risks of respiratory failure and 3-year mortality were similar among the bDMARD, nbDMARD, and other group. For the patients with BROS who used bDMARDs, the risks of NTM and tuberculosis were increased. NTM infection is associated with higher rates of respiratory failure and mortality and sometimes with *P. aeruginosa* or fungal coinfection, causing more frequent exacerbations in patients with bronchiectasis (33, 34). The use of bDMARDs should be cautious in patients with BROS to avoid aggravating the vicious cycle of bronchiectasis.

The study has some limitations. First, although the RA diagnoses were made by rheumatologists on the basis of clinical symptoms and RA serological tests (RF, anti-CCP), not all patients underwent RA serological tests. Second, RA severity score (DAS-28) was recorded in texts in the medical records and was not retrieved in this study. However, the use of bDMARDs in Taiwan is strictly regulated and could thus represent RA with more severe disease activity and physical damage. Third, not all patients underwent sputum culture tests at baseline or during follow-up. Fourth, this study applied an observational design in which data were obtained from a multi-institutional database; thus, treatment selection bias might have occurred during the evaluation of the effects of bDMARDs and nbDMARDs. Fifth, we did not retrieve the detailed reports of high resolution computed tomography. It is possible that these patients may have concurrent RA-related lung disease in addition to bronchiectasis and we could not adjust these confounding factors for respiratory failure and mortality. Sixth, CGRD is a database based on real word practice and it did not include all the required parameters to calculate bronchiectasis severity index.

## Conclusions

This study investigated the clinical outcomes of BROS in an Asian cohort. The severity of exacerbations, levels of RF autoantibodies, and chronology of RA diagnosis were all associated with mortality. The use of bDMARDs to treat patients with BROS should be approached with caution because bDMARDs can increase the risk of tuberculosis and NTM infection in such patients. Future research is necessary to develop multidisciplinary treatments in order to improve the clinical outcomes in patients with BROS.

## Data availability statement

The datasets presented in this article are not readily available to comply with the regulations of Chang Gung Memorial Hospital IRB for patient confidentiality. Requests to access the datasets should be directed to the corresponding author(s).

## Ethics statement

The studies involving human participants were reviewed and approved by the Institutional Review Board of Chang Gung Memorial Hospital approved this study (IRB number: 201800712B0C502). Written informed consent for participation was not required for this study in accordance with the national legislation and the institutional requirements.

## Author contributions

Conceptualization: HY-H, H-CL, and M-HH. Investigation: Y-FF and C-YL: Methodology. Y-TH, J-LH, and T-WL. Data curation: C-HC and C-HL. Validation: H-YH and C-HW. Writing–original draft preparation: H-YH and M-HH. Writing–review and editing: H-CL. All authors contributed to the article and approved the submitted version.

## Funding

This work was supported by Chang Gung Memorial Hospital Research Project Grant (CMRPG3H0931 and CMRPG3K2061), Saint Paul's Hospital Research Project (SPMRP-U1-3001), and the Maintenance Project of the Center for Big Data Analytics and Statistics (Grant CLRPG3D0049) at Chang Gung Memorial Hospital.

## Conflict of interest

The authors declare that the research was conducted in the absence of any commercial or financial relationships that could be construed as a potential conflict of interest.

## Publisher's note

All claims expressed in this article are solely those of the authors and do not necessarily represent those of their affiliated organizations, or those of the publisher, the editors and the reviewers. Any product that may be evaluated in this article, or claim that may be made by its manufacturer, is not guaranteed or endorsed by the publisher.
