# Supplementary material for: Clinical outcomes and prognostic factors of bronchiectasis rheumatoid overlap syndrome: A multi-institution cohort study
Source: Front Med (Lausanne). 2022 Oct 13;9:1004550. doi: 10.3389/fmed.2022.1004550 (PMC9606566; doi:10.3389/fmed.2022.1004550)
Supplement: Supplementary file 1 [file Data_Sheet_1.docx]

**Supplementary data**

**Clinical outcomes and prognostic factors of bronchiectasis rheumatoid overlap syndrome: a multi-institution cohort study**

***Horng-Chyuan Lin ^1, 2^, Hung-Yu Huang ^1,2,3^, Chun-Yu Lin^1, 2^, Yueh-Fu Fang^1, 2^, Chiung-Hung Lin^1, 2^, Yu-Tung Huang ^4^, Chiung-Hsin Chang^1, 2^, Chun-Hua Wang ^1, 2^, Jhen-Ling Huang^4^, Ting-Wei Liao^4^,*** ***and Meng-Heng Hsieh ^1, 2^***

Table S1 Characteristics and outcomes of BROS grouped with RA medication

| **Variable** | bDMARD | nbDMARD | Others | p-value |
| --- | --- | --- | --- | --- |
|  | (n = 27) | (n = 210) | (n = 106) |  |
| **Age** | 63.06 ± 9.10 | 65.31 ± 11.55 | 66.28 ± 11.95 | 0.445 |
| **Gender** |  |  |  | 0.516 |
| Female | 20 (74.07) | 166 (79.05) | 78 (73.58) |  |
| Male | 7 (25.93) | 44 (20.95) | 28 (26.42) |  |
| **Pulmonary function** |  |  |  | 0.006 |
| FEV1 > 80% | 12 (66.67) | 42 (28.77) | 28 (40.00) |  |
| FEV1 50~80% | 1 ( 5.56) | 30 (20.55) | 9 (12.86) |  |
| FEV1 < 50% | 2 (11.11) | 9 ( 6.16) | 10 (14.29) |  |
| FVC < 80% | 3 (16.67) | 65 (44.52) | 23 (32.86) |  |
| **BACI index** | 7.93 ± 3.95 | 9.03 ± 5.36 | 8.08 ± 5.88 | 0.212 |
| **Comorbidity** |  |  |  |  |
| Solid malignancy | 1 ( 3.70) | 8 ( 3.81) | 3 ( 2.83) | 0.903 |
| Liver disease | 5 (18.52) | 34 (16.19) | 21 (19.81) | 0.719 |
| Diabetes | 4 (14.81) | 35 (16.67) | 22 (20.75) | 0.612 |
| Chronic renal disease | 4 (14.81) | 24 (11.43) | 21 (19.81) | 0.132 |
| Hematological malignancy | 0 (0) | 8 ( 3.81) | 3 ( 2.83) | 0.552 |
| Stroke | 3 (11.11) | 26 (12.38) | 17 (16.04) | 0.624 |
| **RA markers** |  |  |  |  |
| Anti-CCP | 195.3 ± 168.8 | 77.04 ± 129.1 | 2.82 ± 1.89 | 0.112 |
| RF value | 211.5 ± 335.7^＊^ | 234.4 ± 766.4 | 106.3 ± 253.7 | <.0001 |
| C-reactive protein | 19.47 ± 30.57 | 39.65 ± 61.26 | 38.05 ± 54.21 | 0.408 |
| ESR | 41.73 ± 25.60^＊^ | 41.36 ± 30.93 | 27.78 ± 25.75 | <.001 |
| **Gap years^1^** | 5.42 ± 3.31^＊^ | 3.20 ± 3.04 | 3.35 ± 3.08 | 0.004 |
| **Sputum microbiology** |  |  |  |  |
| NTM | 8 (50.00) | 37 (30.58) | 13 (26.00) | 0.19 |
| Tuberculosis | 2 (12.50) | 3 (2.48) | 0 (0.00) | 0.05 |
| Pseudomonas | 2 (12.50)＊ | 44 (36.36) | 12 (24.00) | 0.07 |
| **Previous AE (1-year)** | 0.70 ± 1.38 | 1.02 ± 2.10 | 1.07 ± 2.03 | 0.785 |
| Hospitalization | 0.11 ± 0.32 | 0.30 ± 0.82 | 0.27 ± 0.71 | 0.602 |
| Emergency room visit | 0.11 ± 0.32 | 0.49 ± 1.19 | 0.59 ± 1.41 | 0.199 |
| Clinic | 0.52 ± 1.22^＃,＊^ | 0.29 ± 1.40 | 0.23 ± 1.17 | 0.068 |
| **Respiratory failure (1-year)** | 4 (14.81) | 26 (12.38) | 11 (10.38) | 0.780 |
| **Respiratory failure (3-year)** | 6 (22.22) | 48 (22.86) | 14 (13.21) | 0.121 |
| **Death (1-year)** | 4 (14.81) | 21 (10.00) | 16 (15.09) | 0.374 |
| **Death (3-year)** | 6 (22.22) | 51 (24.29) | 31 (29.25) | 0.580 |
| **Death cause** |  |  |  | 0.740 |
| Cardiovascular | 0 (0.00) | 9 (17.65) | 5 (16.13) |  |
| Lung infection | 1 (16.67) | 18 (35.29) | 9 (29.03) |  |
| Malignancy | 0 (0.00) | 4 (7.84) | 3 (9.68) |  |
| RA associated | 2 (33.33) | 4 (7.84) | 3 (9.68) |  |
| Other | 3 (50.00) | 16 (31.37) | 11 (35.48) |  |

Note: AE: acute exacerbation, Anti-CCP: anti-cyclic citrullinated peptide antibody, BACI: bronchiectasis aetiology comorbidity index, BROS: bronchiectasis rheumatoid overlap syndrome, DMARD: disease-modifying antirheumatic drugs, ESR: erythrocyte sedimentation rate, FEV1: forced expiratory volume in one second, FVC: forced vital capacity, NTM, non-tuberculosis mycobacteria, RA: rheumatoid arthritis, RF: rheumatoid factor

1. Gap between RA & bronchiectasis, years

＊ bDMARD vs Others, p<0.05

＃ bDMARD vs nbDMARD, p<0.05

Table S2 Positive rates of sputum microbiology of BROS with or without severe exacerbation during 3 years follow-up

| **General** |  |  |
| --- | --- | --- |
| **microbiology** | **positive/numbers of patients with specimen** | **positive rate (%)** |
| NTM | 40/113 | 35.40 |
| Fungus | 12/33 | 36.36 |
| *Pseudomonas aeruginosa* | 19/87 | 21.84 |
| *Haemophillus influenzae* | 14/87 | 16.09 |
| *Staphylococcus aureus* | 9/87 | 10.34 |
| *Streptococcus pneumoniae* | 8/87 | 9.20 |
| *Klebsiella pneumoniae* | 6/87 | 6.90 |
| Tuberculosis | 4/113 | 3.54 |
| **Severe** |  |  |
| **microbiology** | **positive/numbers of patients with specimen** | **positive rate (%)** |
| NTM | 18/76 | 23.68 |
| Fungus | 11/27 | 40.74 |
| *Pseudomonas aeruginosa* | 37/82 | 45.12 |
| *Stenotrophomonas* | 15/82 | 18.29 |
| *Acinetobacter baumannii* | 10/82 | 12.20 |
| *Staphylococcus aureus* | 10/82 | 12.20 |
| *E. coli* | 9/82 | 10.98 |
| *Klebsiella pneumoniae* | 9/82 | 10.98 |
| Tuberculosis | 1/76 | 1.32 |

Note: BROS: bronchiectasis rheumatoid overlap syndrome, NTM, non-tuberculosis mycobacteria

Table S3 Percentage of missing/not available data of clinical characters of BROS grouped with diagnosis chronology

|  | Total | RA first | BR first |  |
| --- | --- | --- | --- | --- |
|  | (n=343) | (n = 230) | (n = 113) |  |
| **Pulmonary function** | 109 (31.78) | 81 (35.22) | 28 (24.78) |  |
| **RA markers** |  |  |  |  |
| Anti-CCP | 311 (90.67) | 209 (90.87) | 102 (90.27) |  |
| RF value | 89 (25.95) | 55 (23.91) | 34 (30.09) |  |
| C-reactive protein | 30 (8.75) | 17 (7.39) | 13 (11.50) |  |
| ESR | 36 (10.50) | 24 (10.43) | 12 (10.62) |  |
| **Tuberculosis/NTM culture** | 154 (44.90) | 92 (40.00) | 62 (54.87) |  |
| **Fungus culture** | 283 (82.51) | 183 (79.57) | 100 (88.50) |  |
| **Bacterial culture** | 174 (50.73) | 102 (44.35) | 72 (63.72) |  |

Note: Anti-CCP: anti-cyclic citrullinated peptide antibody, BROS: bronchiectasis rheumatoid overlap syndrome, ESR: erythrocyte sedimentation rate, NTM, non-tuberculosis mycobacteria, RA: rheumatoid arthritis, RF: rheumatoid factor
